# Frailty impact on postoperative complications and early mortality rates in patients undergoing radical cystectomy for bladder cancer: a systematic review

**DOI:** 10.1080/2090598X.2020.1841538

**Published:** 2020-11-02

**Authors:** Paola I. Ornaghi, Luca Afferi, Alessandro Antonelli, Maria A. Cerruto, Livio Mordasini, Agostino Mattei, Philipp Baumeister, Giancarlo Marra, Wojciech Krajewski, Andrea Mari, Francesco Soria, Benjamin Pradere, Evanguelos Xylinas, Alessandro Tafuri, Marco Moschini

**Affiliations:** a Department of Urology, Luzerner Kantonsspital, Lucerne, Switzerland; b Department of Urology, University of Verona, Azienda Ospedaliera Universitaria Integrata Verona, Verona, Italy; c Division of Urology, Department of Surgical Sciences, University of Turin, Turin, Italy; d Department of Urology and Oncologic Urology, Wrocław Medical University, Wroclaw, Poland; e Department of Urology, Careggi Hospital, University of Florence, Florence, Italy; f Department of Urology, CHRU Tours, Francois Rabelais University, Tours, France; g Department of Urology, Bichat-Claude Bernard Hospital, Assistance Publique-Hôpitaux De Paris, Paris University, Paris, France

**Keywords:** Bladder cancer, cystectomy, frailty, complications, mortality

## Abstract

**Objective**: To assess the prevalence of frailty, a status of vulnerability to stressors leading to adverse health events, in bladder cancer patients undergoing radical cystectomy (RC), and test the impact of frailty measurements on postoperative adverse outcomes.

**Methods**: A systematic review of English-language articles published up to April 2020 was performed. Electronic databases were searched to quantify the frailty prevalence in RC patients and assess the predictive ability of frailty indexes on RC-related outcomes as postoperative complications, early mortality, hospitalization length (LOS), costs, discharge dispositions, readmission rate.

**Results**: Eleven studies were selected. Patients’ frailty was identified by Johns Hopkins indicator (JHI) in two studies, 11-item modified Frailty Index (mFI) in four, 5-item simplified FI (sFI) in three, 15-point mFI in one, Fried Frailty Criteria in one. Considering all the frailty measurements applied, 8% and 31% of patients were frail or pre-frail, respectively. Frail (43%) and pre-frail patients (35%) were more at risk of major complications compared to non-frail (27%) using sFI; with JHI the percentages of frail and non-frail were 53% versus 19%. According to JHI and mFI frailty was related to longer LOS and higher costs. JHI identified that 3% of frail patients experience in-hospital mortality versus 1.5% of non-frail. Finally, using sFI, frail (28%), and pre-frail (19%) were more likely to be discharged non-home compared to non-frail patients (8%) and had a higher risk of 30-day mortality (4% and 2% versus 1%).

**Conclusions**: Almost half of RC patients were frail or pre-frail, conditions significantly related to an increased risk of postoperative adverse events with higher rates of major complications and early mortality. The most-used frailty index was mFI, while JHI and sFI resulted the most reliable to predict early postoperative RC-related adverse outcomes and should be routinely included in clinical practice after better standardization throughout prospective comparative studies.

**Abbreviations**: ACG: Adjusted Clinical Groups; ACS: American College Surgeons; AUC: area under the curve; BCa: bladder cancer; CCI: Charlson Comorbidity Index; CSHA-FI: Canadian Study of Health and Aging Frailty Index; CCS: Clavien-Dindo Classification Score; ERAS: Enhanced Recovery After Surgery; FFC: Fried Frailty Criteria; (e)(m)(s)FI: (extended) (modified) (simplified) Frailty Index; ICU: intensive care unit; IQR: interquartile range; (p)LOS: (prolonged) length of hospital stay; NSQIP: National Surgical Quality Improvement Program; OR: odds ratio; (O)PN: (open) partial nephrectomy; PRISMA: Preferred Reporting Items for Systematic reviews and Meta-Analyses; (O)(RA)RC: (open)(robot-assisted) radical cystectomy; (O)RN: (open) radical nephrectomy; ROC: receiver operating characteristic; RNU: radical nephroureterectomy; (R)RP: (retropubic) radical prostatectomy; RR: relative risk; THCs: total hospital charges; nephrectomy; UD: urinary diversion

## Introduction

Bladder cancer (BCa) is the second most common genitourinary malignancy, with 81 400 new cases and 17 980 deaths estimated in 2020 in the United States [1]. Radical cystectomy (RC) with urinary diversion (UD) is considered the standard treatment for localised muscle-invasive BCa and recurrent high-grade non-muscle-invasive BCa [2,3]. The bilateral pelvic lymph node dissection plays, especially in high-risk patients, an essential role in this procedure [4]. RC is a highly complex intervention associated with a high risk of postoperative complications and adverse oncological and functional outcomes [5]. This occurs despite improvements in the quality of care provided by the introduction of minimally invasive surgical techniques [6,7], and the implementation of multimodal protocols, such as Enhanced Recovery After Surgery (ERAS) programmes, which aim to improve and accelerate patient recovery [8].

The incidence of BCa increases with age. Thus, in developed countries, where life expectancy is progressively increasing, the number of elderly patients with BCa is expected to further expand in the future [9,10]. However, chronological age is an unreliable indicator of patients’ health status, whilst the assessment of frailty is considered as a more accurate method of evaluation, and has become increasingly recognised as one of the most important issues in healthcare and health outcomes [11]. Frailty is a predominantly geriatric condition that can be closely related to malnutrition, low activity, and catabolic status; it is a multifactorial syndrome characterised by a declining strength and endurance that induce a reduction of physiological reserve, while increasing the vulnerability to stressors and predisposing to a higher risk of adverse events and/or death [12,13]. Frailty has a meaningful impact in oncological patients, as it has been shown to reduce physical reserves and compromise recovery after stressful events like surgery or systemic treatment [11]. Therefore, several frailty scores have been introduced to specifically assess this condition and to comprehensively predict the risk of unfavourable outcomes in candidates for major surgery [10,14]. To date, the impact of frailty on postoperative RC outcomes has not yet been thoroughly explored. Moreover, although several methods have been developed to measure patient’s frailty, the best in terms of both predictive ability and ease-of-use has not yet been identified.

The present systematic review aimed to quantify the prevalence of frailty in patients with BCa treated with RC and comprehensively summarise the current evidence on the prognostic role of preoperative frailty measurements to verify their impact on postoperative complications and early mortality, along with other endpoints such as length of hospital stay (LOS), hospitalisation costs, type of discharge disposition and rate of hospital readmission.

## Materials and methods

### Literature search strategy and study selection

A systematic review of the English-language literature published until 1 April 2020 was performed scrutinising the Medical Literature Analysis and Retrieval System Online (MEDLINE), the Excerpta Medica dataBASE (EMBASE), and Web of Science databases according to the Preferred Reporting Items for Systematic reviews and Meta-Analyses (PRISMA) statement [15]. The research was performed using the following search string: *(‘cystectomy’ OR ‘radical cystectomy’ OR ‘bladder cancer’) AND (‘frailty’ OR ‘frail’) AND (‘mortality’ OR ‘morbidity’ OR ‘complications’ OR ‘length of stay’ OR ‘survival’ OR ‘readmission’)*. According to the aim of the present study, all eligible texts reporting the postoperative outcomes under examination in patients screened by a preoperative frailty indicator that were treated with RC for BCa were included in the systematic review. After a first screening based on study title and abstract, all articles were examined based on full-text and excluded with reasons when inappropriate. The following types of articles were excluded from the systematic review: review articles, case reports, editorial/author replies or comments to other articles, and studies that dealt with research unrelated to our topic.

### Outcomes of interest

Our primary outcomes were postoperative complications and early mortality rates; secondary endpoints were LOS, costs of care and total hospital charges (THCs), different kinds of discharge disposition, and unplanned readmission rate. Complications were defined as any postoperative event caused by surgery, within 30 or 90 days after RC, altering the normal postoperative course and/or delaying discharge; the major complications were graded according to the Clavien–Dindo Classification Score (CCS) [16], or to the American College Surgeons–National Surgical Quality Improvement Program (ACS-NSQIP). Non-home discharge was defined as any discharge disposition different than at home (rehabilitation, skilled or unskilled nursing facility, short-term hospital); early mortality was defined as any-cause death occurring during post-surgical hospital stay (in-hospital mortality) or within 30 days after surgery.

### Frailty indexes and assessments

In the present systematic review, frailty was assessed based on scores that evaluate either clinical indexes detecting the presence of comorbidity and impaired functional status or patients’ physiological fitness among studies analysing patients with BCa treated with RC. The indexes have been adapted from the original 70-item Canadian Study of Health and Aging Frailty Index (CSHA-FI), which estimated frailty across 70 possible clinical deficits, ranging from the presence and severity of current diseases, ability in the activities of daily living, and physical and neurocognitive health status [17], while frailty assessments included the Fried Frailty Criteria (FFC) [18], which evaluated grip strength, gait speed, feelings of exhaustion, physical activity level, and shrinking. From the CSHA-FI derived a validated, binary measure called the Johns Hopkins Adjusted Clinical Groups (ACG) frailty-defining diagnoses indicator [19], which was developed specifically to be applied to health administrative data and evaluated frailty using 10 clusters of diagnoses. The other frailty indexes used were also based on CSHA-FI, but they have an accumulated score that classifies a patient into different degrees of frailty, with value thresholds varying based on total criteria within the index; they were a CSHA-FI reduced form, called the 11-item modified Frailty Index (mFI) or extended Frailty Index (eFI), and an easier-to-use five-item simplified Frailty Index (sFI), which had a correlation >0.9 compared to the 11-item mFI; it only included variables that provided comprehensive information about body’s function and had the highest predictive potential [20]. Another indicator used was the 15-point modified Frailty Index (15-point mFI); it was developed by adding to the mFI four ACS-NSQIP variables related to oncology patients [21]. The items and criteria for the frailty measurements are summarised in Table 1.

**Table 1.** Selection of frailty indexes and assessments used in patients with BCa treated with RC

Frailty metric	Measurement items and criteria	
Canadian Study of Health and Aging frailty index (CSHA-FI)	Assessment of 70 variables measuring the presence and severity of current disease, ability for ADL, and physical and neurological signs from the clinical examination of mobility, function, and self-rated health	
	*Index = total number of deficits identified/total number of deficits measured*	
Johns Hopkins Adjusted Clinical Groups (ACG) frailty-defining diagnoses indicator	1. Malnutrition	Nutritional marasmus
		Other severe protein-calorie malnutrition
	2. Dementia	Senile dementia with delusional or depressive features
		Senile dementia with delirium
	3. Severe vision impairment	Profound impairment, both eyes
		Better eye: moderate or severe impairment, Lesser eye: profound
	4. Decubitus ulcer	Decubitus ulcer
	5. Incontinence of urine	Incontinence without sensory awareness
		Continuous leakage
	6. Loss of weight	Abnormal loss of weight and underweight
		Feed difficulties and mismanagement
	7. Fecal incontinence	Incontinence of feces
	8. Social support needs	Lack of housing
		Inadequate housing
		Inadequate material resources
	9. Difficulty in walking	Difficulty in walking
		Abnormality of gait
	10. Fall	Fall on stairs or steps
		Fall from wheelchair
	*Patients defined as frail: ≥1 of the 10 items*	
11-item modified Frailty Index (mFI)	1. Functional health status before surgery (partially/totally dependent)	
	2. Impaired sensorium	
	3. Diabetes mellitus type II	
	4. Chronic obstructive pulmonary disease	
	5. Congestive heart failure exacerbation within 30 days before surgery	
	6. History of myocardial infarction within 6 months before surgery	
	7. Hypertension	
	8. Prior cardiac surgery, percutaneous coronary intervention, or angina within 1 month before surgery	
	9. History of transient ischaemic attack	
	10. History of cerebrovascular accident	
	11. Peripheral vascular disease requiring surgery or active claudication present	
	*Index = number of factors present/11; Scored as: robust: 0–0.09; pre-frail: 0.09–0.18; frail: ≥0.27*	
	*Index = risk factor per patient; Scored as 0, 1, 2, or ≥3 risk factors*	
5-item simplified Frailty Index (sFI)	1. Diabetes mellitus	Therapy with oral agents
		Therapy with insulin
	2. Functional status	
	3. Chronic obstructive pulmonary disease	
	4. Congestive heart failure exacerbation within 30 days before surgery	
	5. Hypertension requiring medication	
	*Scored as 0, 1, 2, or ≥3 risk factors*	
15-point modified Frailty Index (mFI)	1–11: 11-item mFI	
	12. Weight loss within the last 6 months >10%	
	13. Chemotherapy or radiation before surgery	
	14. History of metastasis	
	15. Severe renal failure or currently on dialysis	
	*Index = number of factors present/15; Scored as: 0–0.05, 0.05–0.10, 0.10–0.15, 0.15–0.20, >0.20*	
Fried Frailty Criteria (FFC)	1. Grip strength, kg	Measured three times in each hand using a JAMAR hydraulic hand dynamometer.
	2. Gait speed, s	Measured as time to walk 15 feet (4.6 m) at a normal pace.
	3. Physical activity, kcals	Patients were asked ‘Do you do any exercise on a regular basis?’ and if not, then, ‘How many hours per day do you spend walking and/or standing?’
	4. Shrinking	Defined as ≥10 pounds (4.5 kg) of unintentional weight loss in the last year.
	5. Exhaustion	Patients were asked how often in the last week they felt ‘everything I did was an effort’ (#1) and ‘I could not get going’ (#2).
	*Scored as: non-frail: 0–1 points, intermediately frail: 2–3 points, frail: 4–5 points*	

ADL: activities of daily living; ACG: Adjusted Clinical Groups

## Results

### Evidence synthesis

**Figure 1.**
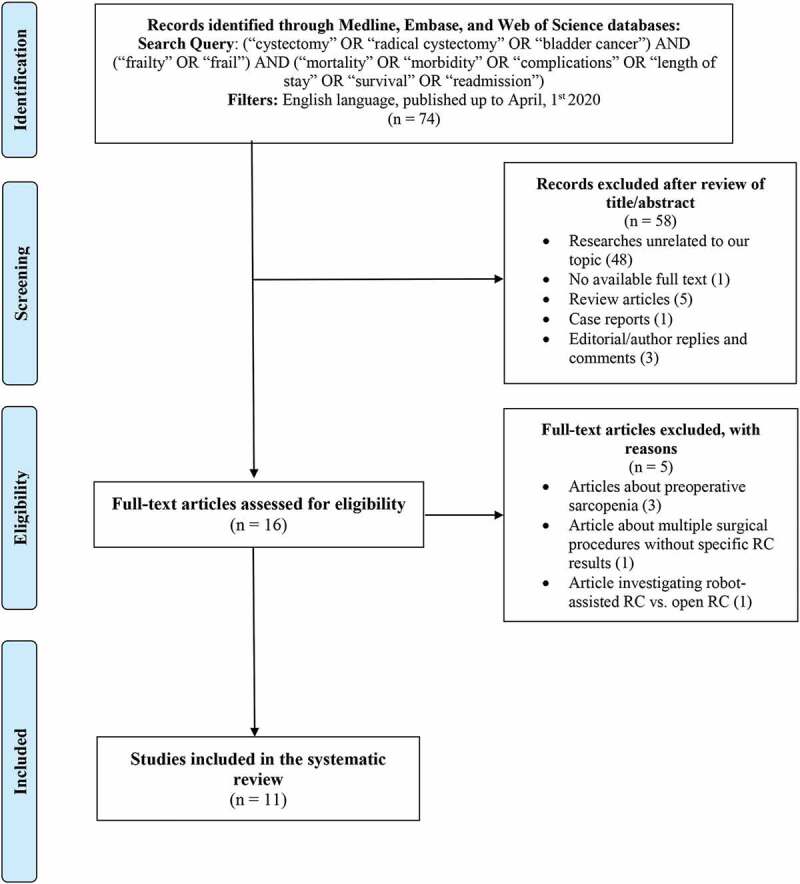
PRISMA flowchart for the article selection process to analyse the impact of preoperative frailty on early postoperative outcomes in patients with BCa treated with RC

Figure 1 reports the flow diagram of the selection process used for this systematic review. From a total of 74 articles screened, 16 were initially assessed for eligibility. Of these, five were subsequently excluded after full-text evaluation, and 11 were selected and critically analysed by the authors.

### Study population and design

Overall, our systematic review included 60 907 patients. The characteristics of the 11 studies selected, frailty indicators, and early postoperative outcomes are reported in Supplementary Table S1 [21–31]. Eligible articles were published between 2015 and 2019 involving patients that underwent RC from 2000 to 2018. In all, 10 of the 11 studies had a retrospective design. Eight retrospective studies used a frailty index based on the available data points in a national database like the National Inpatient Sample database [22], Nationwide Readmissions Database [23] or ACS-NSQIP database [21,24–28]; one study used available data from a single-centre cohort [29] and another study from a multicentre cohort [30]; the only prospective study used a frailty assessment based on data from a single-centre cohort [31].

The method employed to identify frailty was not homogeneous across the different studies. Two studies detected frailty using the Johns Hopkins ACG frailty-defining diagnoses indicator [22,23], four used the 11-item mFI or eFI [24–26,29], two the five-item sFI [28,30], one the sFI and comparing it with the eFI [27], one using the 15-point mFI and comparing it with 11-item mFI [21], and one study using the FFC [31].

Two studies evaluated RC along with other urological surgical procedures such as minimally invasive radical prostatectomy, retropubic radical prostatectomy/radical prostatectomy (RRP/RP), minimally invasive radical nephrectomy (RN), open RN (ORN)/RN, minimally invasive partial nephrectomy, open partial nephrectomy/partial nephrectomy (OPN/PN), and radical nephroureterectomy (RNU) [21,28].

### Prevalence of frailty in patients with BCa treated with RC

Overall, 8% (range 2–24%) of patients with BCa undergoing RC were frail, whereas 31% (range 14–65%) were pre-frail. De Nunzio *et al*. [30] reported a majority of frail (39%) than pre-frail patients (27%) evaluating a population of octogenarian RC patients.

### Surgical approach used for RC

The surgical approach used for RC was open in one study, multiple (open RC [ORC] or minimally invasive RC, including laparoscopic and robot-assisted RC [RARC]) in four studies [22,23,29,31], and was not reported/specified in the remaining six studies. In the studies reporting a multiple surgical approach, the percentage of frail patients undergoing minimally invasive RC was 14% (range 9–18%), while in non-frail patients it was 17% (8–24%) [22,23,29].

### Frailty based on Johns Hopkins ACG frailty-defining diagnoses indicator

Two studies [22,23] used the Johns Hopkins ACG index to explore the association between frailty and postoperative RC outcomes.

#### Postoperative complication rates

Palumbo *et al*. [22] investigated the impact of frailty on the risk of postoperative overall complications finding a higher risk in frail (odds ratio [OR] 1.54, 95% CI 1.44–1.65; *P* < 0.001) compared to non-frail patients. The frailty’s effect overlapped with Charlson Comorbidity Index (CCI) impact (CCI of ≥2: OR 1.54, 95% CI 1.41–1.68; *P* < 0.001) and outperformed age ≥75 years (OR 1.16, 95% CI 1.09–1.23; *P* < 0.001). In the Michel *et al*. [23] study, frailty was the strongest independent predictor of intensive care unit (ICU)-level complications (CCS = IV) (OR 4.74, 95% CI 3.60–6.25; *P* < 0.001) outperforming both CCI (CCI ≥3: OR 2.09, *P* < 0.001) and being aged ≥75 years (OR 1.37, 95% CI 1.12–1.68; *P* = 0.002).

#### LOS and costs

In the Michel *et al*. [23] study, frail patients had a LOS almost twice as long [median (interquartile range, IQR) 15 (9–21) vs 7 (6–10) days, *P* < 0.001] and hospital-related costs 1.5-times as high [median (IQR) $39 665 ($28 196–$56 397) vs $27 307 ($21 145–$36 049), *P* < 0.001) as their non-frail counterparts. Palumbo *et al*. [22] found that frailty was significantly related to the prolongation of LOS [relative risk (RR) 1.32, 95% CI 1.28–1.35; *P* < 0.001], and had a stronger impact than being aged ≥75 years (RR 1.06, 95% CI 1.04–1.09; *P* < 0.001) and having a CCI of ≥2 (RR 1.12, 95% CI 1.08–1.16; *P* < 0.001). Evaluating the role of frailty on THCs, the authors found that a frail status (+$8003.3, 95% CI $6849.1–$9158.2; *P* < 0.001) was a significant predictive factor of higher THCs with a doubled impact compared to a CCI of ≥2 (+$3910.7, 95% CI $2726.9–$5094.5; *P* < 0.001) [22].

#### Discharge disposition

Frail patients were found to be at a higher risk of non-home discharge (i.e. to a nursing facility or short-term hospital) in the study by Michel *et al*. [23] (OR 3.43, 95% CI 2.50–4.69; *P* < 0.001).

#### Unplanned readmission

Michel *et al*. [23] found that the 30-day readmission rate was similar between frail and non-frail patients (31.8% vs 29.3%; +Δ2.5%, 95% CI – 4.2 to 9.2%); however, when readmitted, frail patients had significantly higher costs [median (IQR) $35 732 (26 638–56 440)] of readmission compared to their non-frail counterparts [median (IQR) $29 319 (22 314–39 513)].

### Early mortality rates

Both studies showed a significantly increased risk of in-hospital mortality amongst patients diagnosed as frail compared with non-frail patients. In the Palumbo *et al*. [22] study, the risk of dying before discharge was almost 50% higher (OR 1.45, 95% CI 1.17–1.80; *P* = 0.001), while Michel *et al*. [23] reported an even more than two-fold increase in this unfavourable outcome (OR 2.30, 95% CI 1.08–4.92; *P* = 0.03).

#### Frailty based on modified or simplified forms of the CSHA-FI

Eight studies adopted the CSHA-FI using the 11-item mFI, the five-item sFI, or the 15-point mFI to analyse the role of frailty on early postoperative RC outcomes.

#### Postoperative complication rates

All eight studies investigated the relationship between preoperative frailty and the occurrence of complications after RC. In the study by Sathianathen *et al*. [27], the complication rate was 15% in pre-frail (sFI = 2) and 26% in frail patients (sFI ≥3), while the percentages for patients with sFI = 0 and sFI = 1 were 8% and 10%, respectively (all *P* < 0.001). The authors found that both being pre-frail (OR 1.73, 95% CI 1.32–2.26) and being frail (OR 3.22, 95% CI 2.01–5.17) were stronger predictors of the occurrence of 30-day major complications (CCS ≥III) compared to obesity (OR 1.45, 95% CI 1.13–1.89), smoking habit (OR 1.54, 95% CI 1.24–1.92), and history of a bleeding disorder (OR 1.58, 95% CI 1.05–2.38); being frail had a greater impact even than being underweight (OR 1.93, 95% CI 1.04–3.57). The receiver operating characteristic (ROC) curves showed that the predictive ability of the sFI was better than the ASA score [Area Under the Curve (AUC) 0.561 vs AUC 0.544, *P* = 0.002], but not different from either the eFI (*P* = 0.688) or the NSQIP risk calculator (*P* = 0.163). In the Chappidi *et al*. [26] study both being pre-frail (mFI = 2: OR 1.84, 95% CI 1.28–2.64; *P* = 0.001) and being frail (mFI ≥3: OR 2.58, 95% CI 1.47–4.55; *P* = 0.001) were conditions significantly related to a higher risk of 30-day severe complications (CCS IV–V), these conditions had a stronger impact than being aged ≥80 years (OR 1.58, 95% CI 1.11–2.27; *P* = 0.01). In the Pearl *et al*. [24] study being frail (mFI ≥0.27) was significantly related to an augmented percentage of any (29% vs 20%, *P* < 0.001) and major (19% vs 9%, *P* < 0.001) in-hospital complications compared to being robust (mFI = 0).

De Nunzio *et al*. [30] focussed on a multicentre cohort of 117 patients aged ≥80 years undergoing RC for BCa; in this population, most major complications (CCS ≥III) occurred in frail patients (sFI ≥3) (11% vs 3%, *P* = 0.02) compared to the other patients (sFI <3) and multivariable analysis confirmed that an sFI of ≥3 was an independent predictor of an increased risk of 90-day major complications (OR 3.10, 95% CI 0.70–13.70; *P* = 0.01).

Two studies investigated the impact of frailty in several urological procedures [21,28]. In the Taylor *et al*. [28] study, an increasing sFI was significantly associated with higher rates of 30-day overall complications (sFI = 0: 51%; sFI = 1: 54%; sFI = 2: 61%; and sFI ≥3: 66%; *P* < 0.001) in patients undergoing RC; for major complications the rate was 2–6% lower than for any complication at each sFI, but demonstrated significant increases with rising sFI (45%; 49%; 55%; 60%, respectively; *P* < 0.001). In the study by Lascano *et al*. [21], frail patients (15-point mFI >0.20 vs 0–0.05) undergoing RC had a higher risk of developing a severe complication (CCS = IV) (17% vs 7%, *P* < 0.001) compared to the non-frail counterparts.

In the Meng *et al*. [25] study, the mFI did not achieve significance in predicting neither any adverse events nor serious adverse events after RC. Lastly, in the study by Woldu *et al*. [29], the mFI had a weak association with the occurrence of major complications (CCS ≥III) after surgery (AUC 0.551, 95% CI 0.471–0.631; *P* = 0.2), similarly to the ASA score (AUC 0.535, *P* = 0.4) and the CCI (AUC 0.565, *P* = 0.1).

#### LOS and costs

The impact of frailty on LOS was explored in six studies. In the Pearl *et al*. [24] study being frail (mFI ≥0.27) was significantly correlated with a prolonged LOS (pLOS) [median (IQR) 9 (4–57) vs 7 (0–75) days; *P* < 0.001) compared to being robust (mFI = 0). The Woldu *et al*. [29] analyses showed that a pre-frail (mFI = 2) and frail status (mFI ≥3) were predictors of pLOS compared to non-frail status (mFI = 0) [mean (SD) 8.2 (5.7) and 11.3 (11.1) vs 7.6 (4.7) days; *P* = 0.003], while neither the ASA score (*P* = 0.07) nor the CCI (*P* = 0.1) reached the significance for this association. In their study, the frail group was significantly related to higher costs of operation plus hospitalisation compared to the other groups ($30 354 vs ~22 500, *P* = 0.003). In the study by Chappidi *et al*. [26] the higher frailty group (mFI ≥2) had a significant difference in LOS compared to the lower frailty group (mFI <2) [median (IQR) 11.0 (10.2–11.8) vs 10.0 (9.7–10.4); *P* < 0.01]. In three studies frailty results were not extractable [28] or did not achieve statistical significance in predicting a prolongation of LOS after RC [25,30].

#### Discharge disposition

Four studies evaluated the relationship between frailty and discharge disposition in patients with BCa undergoing RC. Pearl *et al*. [24] found that the likelihood of not being discharged at home, but to a rehabilitation or a nursing facility, was significantly increased in patients diagnosed as pre-frail according to mFI (mFI 0.09–0.18: OR 1.37, 95% CI 1.07–1.74; *P* = 0.01) and more than doubled in patients diagnosed as frail (mFI ≥0.27: OR 2.33, 95% CI 1.34–4.03; *P* = 0.003) compared to non-frail patients. In the Sathianathen *et al*. study [27], the impact of being pre-frail or frail based on the sFI and the risk of being discharged to a facility instead of home was almost overlapping with the Pearl *et al*. [24] results [pre-frail patients sFI = 2: OR 1.54, 95% CI 1.18–2.02; frail patients sFI ≥3: OR 2.31, 95% CI 1.40–3.82]. Moreover, the sFI proved to be a comparable predictor of non-home discharge than both the ASA score (*P* = 0.4) and the more complex eFI (*P* = 0.5). In two studies frailty results were not extractable [28], or did not achieve statistical significance in predicting non-home discharge after RC [25].

#### Unplanned readmission

The association between a frail status and the rate of readmission after RC was analysed in four studies. In three studies frailty was not significantly related to this outcome [21,26,29] and in one study results related to RC were not extractable [28].

### Early mortality rates

Four studies analysed the impact of a frail status on early mortality rates. Chappidi *et al*. [26] found that frail and pre-frail patients (mFI ≥2) were more likely to die within 30 days of RC than the other patients (3.5% vs 1.8%, *P* = 0.01). In the study by Taylor *et al*. [28], an increasing sFI was associated with increased rates of 30-day mortality (sFI = 0: 1%; sFI = 1: 1.5%; sFI = 2: 2%; and sFI ≥3: 4%; *P* < 0.001) in patients undergoing RC. Lascano *et al*. [21] found that being frail (15-point mFI >0.20) was associated with a higher risk of 30-day mortality compared to not being frail (15-point mFI: 0–0.05; 7% vs 2%; *P* = 0.005). In one study the association between frailty and 30-day mortality did not achieve statistical significance [25].

#### Frailty based on fried frailty criteria

A single article investigated the impact of frailty on early postoperative RC outcomes using the FFC. Burg *et al*. [31], in their prospective study, enrolled patients aged ≥65 years elected for RC and preoperatively assessed and classified them using the FFC. Overall, 89% of the patients had a full assessment according to the FFC; among them, 40% were intermediately frail (FFC 2–3) and 6% were frail (FFC 4–5). The authors found that ‘shrinking’ was a predictor of a higher rate of overall 30-day complications (OR 3.79, 95% CI 1.64 − 9.26; *P* = 0.002), and being intermediately frail or frail was associated with higher rates of major (CCS ≥III) 30-day (OR 4.87, 95% CI 1.39 − 22.77; *P* = 0.02) and 90-day complications (OR 3.01, 95% CI 1.05 − 9.37; *P* = 0.04) compared to being non-frail (FFC 0–1). Moreover, decreased gait speed was a predictor of 90-day major complications (OR 1.70, 95% CI 1.05–2.89; *P* = 0.04) and was significantly related to a higher 90-day readmission risk (*P* = 0.02). Finally, physical activity was protective for both 30-day major complication rate (OR 0.36, 95% CI 0.12 − 0.78; *P* = 0.04) and 90-day any complication rate (OR 0.84, 95% CI 0.69−1.00; *P* = 0.03).

#### Comparisons between frailty measurements

An overview of the results extracted from the collected studies and grouped by endpoints of interest is shown in Table 2 [21–31].

**Table 2.** Overview of the studies investigating the relationship between preoperative frailty and early postoperative RC-related outcomes, grouped by endpoints of interest

Reference	Study design	Study size, *n*	Type of surgery	Preoperative frailty indicator(s)	Postoperative outcome(s)	Findings
Postoperative complication rates						
Palumbo *et al*., 2019 [22]	Retrospective (NIS database)	23,967	ORC, LRC, RARC	Johns Hopkins ACG frailty-defining diagnoses indicator	Overall complications	Frail patients vs non frail: 67.9% vs 55.8%, *P* < 0.001 Frail patients vs non-frail: OR 1.54 (95% CI 1.44–1.65), *P* < 0.001
Michel *et al*., 2019 [23]	Retrospective (NRD)	9459	ORC, RARC	Johns Hopkins ACG frailty-defining diagnoses indicator	ICU-level complications (CCS = IV)	Frail patients vs non-frail: 52.9% vs 18.6%, +Δ34.3% (95% CI 30.0–40.6%), *P* < 0.001 Frail patients vs non-frail: OR 4.74 (95% CI 3.60–6.25), *P* < 0.001
Pearl *et al*., 2017 [24]	Retrospective (ACS-NSQIP Database)	4330	RC	11-item mFI^a^	Any in-hospital complication; Major in-hospital complications^e^	mFI, any in-hospital complication: frail (mFI ≥0.27), 28.7%; pre-frail (mFI 0.09–0.18), 24.8%; robust (mFI = 0),19.6%; *P* < 0.001 mFI, major in-hospital complications: frail, 19.2%; pre-frail, 12.6%; robust, 8.8%; *P* < 0.001
Meng *et al*., 2018 [25]	Retrospective (ACS-NSQIP Database)	1516	RC	11-item mFI^a^	30-day any adverse events; 30-day serious adverse events	mFI, 30-day any adverse events: AUC 0.500, 95% CI (0.471–0.529), did *not* reach statistical significance mFI, 30-day serious adverse events: AUC 0.516, 95% CI (0.483–0.550), did *not* reach statistical significance
Woldu *et al*., 2018 [29]	Retrospective (monocentric study)	346	ORC, RARC	11-item mFI^b^	30-day major complications (CCS ≥III)	mFI: AUC 0.551, 95% CI (0.471–0.631), *P* = 0.2
Chappidi *et al*., 2016 [26]	Retrospective (ACS-NSQIP Database)	2679	RC	11-item mFI^b^	30-day severe complications (CCS IV–V)	Frail and pre-frail patients (mFI ≥2) vs the others (mFI <2): 14.6% vs 8.3%, *P* < 0.001 Pre-frail patients (mFI = 2) vs non-frail (mFI = 0): OR 1.84, 95% CI (1.28–2.64), *P* = 0.001 Frail patients (mFI ≥3) vs non-frail (mFI = 0): OR 2.58, 95% CI (1.47–4.55), *P* = 0.001
Sathianathen *et al*., 2018 [27]	Retrospective (ACS-NSQIP Database)	5516	RC	5-item sFI^c^ and 11-item eFI^b^	30-day major complications (CCS ≥III)	Frail (sFI ≥3) and pre-frail patients (sFI = 2) vs non-frail (sFI = 0): 26.0% and 15.1% vs 7.7%, *P* < 0.001. Pre-frail patents vs non-frail: OR 1.73, 95% CI (1.32–2.26) Frail patients vs non-frail: OR 3.22, 95% CI (2.01–5.17) sFI: AUC 0.56, 95% CI (0.47–0.57) eFI: AUC 0.56, *P* = 0.7
De Nunzio *et al*., 2019 [30]	Retrospective (multicentre study)	117, aged ≥80 years	RC	5-item sFI^c^	90-day major complications (CCS ≥III)	Frail patients (sFI ≥3) vs other patients (sFI <3): 11.1% vs 3.4%, *P* = 0.02 Frail patients vs other patients: OR 3.10, 95% CI (0.70–13.70), *P* = 0.01
Taylor *et al*., 2019 [28]	Retrospective (ACS-NSQIP Database)	92,999 (3823,8154, 14,668, 2817, 13,953, 5678, 9466)	MIRP, RRP, MIRN, ORN, MIPN, OPN, RC	5-item sFI^d^	30-day any complications; 30-day major complications^e^	In RC patients: sFI 30-day any complication: sFI = 0, 50.8%; sFI = 1, 54%; sFI = 2, 61.2%; and sFI ≥3, 66.4%; *P* < 0.001 sFI 30-day major complications: sFI = 0, 45.3%; sFI = 1, 49.0%; sFI = 2, 55.3%; and sFI ≥3 60.3%; *P* < 0.001
Lascano *et al*., 2015 [21]	Retrospective (ACS-NSQIP Database)	41,681 (5709, 7791 1443, 23,350,3388)	PN, RN, RNU, RP, ORC	15-point mFI and 11-item mFI^a^	30-day CCS = IV complications	In RC patients: Frail patients (15-point mFI ≥0.20) vs non frail (15-point mFI 0–0.05): 17.0% vs 6.6%, *P* < 0.001 15-point mFI: AUC 0.585, *P* < 0.001 The 15-point mFI was superior to the 11-item mFI in all the comparisons
Burg *et al*., 2018 [31]	Prospective (monocentric study)	123, aged ≥65 years	ORC, RARC	FFC	30/90-day any complications; 30/90-day major complications (CCS ≥III)	Shrinking, any 30-day complication: OR 3.79 (95% CI 1.64–9.26), *P* = 0.002 Physical activity, 30-day major complications: OR 0.36 (95% CI 0.12 − 0.78), *P* = 0.04 Physical activity, 90-day any complication: OR 0.84 (95% CI 0.69–1.00), *P* = 0.03 Intermediately frail or frail vs non-frail, 30-day major complications: OR 4.87 (95% CI 1.39–22.77), *P* = 0.02 Intermediately frail or frail vs non-frail, 90-day major complications: OR 3.01 (95% CI 1.05–9.37), *P* = 0.04
LOS and costs						
Palumbo *et al*., 2019 [22]	Retrospective (NIS database)	23,967	ORC, LRC, RARC	Johns Hopkins ACG frailty-defining diagnoses indicator	pLOS; THCs	Frail patients vs non-frail, pLOS: RR 1.32 (95% CI 1.28–1.35), *P< *0.001 Frail patients vs non-frail, THCs: +$8003.3 (95% CI 6849.1–9158.2), *P< *0.001)
Michel *et al*., 2019 [23]	Retrospective (NRD)	9459	ORC, RARC	Johns Hopkins ACG frailty-defining diagnoses indicator	LOS; Hospital-related costs	Frail patients vs non-frail, LOS: median (IQR) 15 (9–21) vs 7 (6–10) days, *P< *0.001 Frail patients vs non-frail, LOS: OR 0.58 (95% CI 0.50–0.66), *P< *0.001 Frail patients vs non-frail, hospital-related costs: median (IQR) $39 665 ($28 196–$56 397) vs $27 307 ($21 145–$36 049), *P< *0.001 Frail patients vs non-frail, hospital-related costs: OR 0.42 (95% CI 0.34–0.49), *P< *0.001
Pearl *et al*., 2017 [24]	Retrospective (ACS-NSQIP Database)	4330	RC	11-item mFI^a^	LOS	Frail patients (mFI ≥0.27) vs robust (mFI = 0): 9 (4–57) vs 7 (0–75) days, *P< *0.001
Meng *et al*., 2018 [25]	Retrospective (ACS-NSQIP Database)	1516	RC	11-item mFI^a^	pLOS (>75th percentile)	mFI: AUC 0.529 (95% CI 0.498–0.560), did *not* reach statistical significance
Woldu *et al*., 2018 [29]	Retrospective (monocentric study)	346	ORC, RARC	11-item mFI^b^	LOS; Hospital-related costs	Frail (sFI ≥3) and pre-frail patients (sFI = 2) vs non-frail (sFI = 0), mean (SD) LOS: 11.3 (11.1) and 8.2 (5.7) vs 7.6 (4.7) days, *P* = 0.003 Frail patients (mFI ≥3) vs other groups, hospital-related costs: $30 354 vs ~$22 500, *P* = 0.003
Chappidi *et al*., 2016 [26]	Retrospective (ACS-NSQIP Database)	2679	RC	11-item mFI^b^	LOS	Pre-frail and frail patients (mFI ≥2) vs other patients (mFI <2): 11.0 (10.2–11.8) vs 10.0 (9.7–10.4) days, *P* < 0.01
De Nunzio *et al*., 2019 [30]	Retrospective (multicentre study)	117, aged ≥80 years	RC	5-item sFI^c^	LOS	Frail patients (sFI ≥3) vs other patients (sFI <3): 16 (10–23) vs 13 (10–20) days, *P* = 0.28
Discharge disposition						
Michel *et al*., 2019 [23]	Retrospective (NRD)	9459	ORC, RARC	Johns Hopkins ACG frailty-defining diagnoses indicator	Non-home vs home discharge	Frail patients vs non-frail: 33.9% vs 11.6%, +Δ22.2% (95% CI 16.2–14.5%), *P* < 0.001 Frail patients vs non-frail: OR 3.43 (5% CI 2.50–4.69), *P* < 0.001
Pearl *et al*., 2017 [24]	Retrospective (ACS-NSQIP Database)	4330	RC	11-item mFI^a^	Discharge to non-home vs home care	Frail patients (mFI ≥0.27) vs robust (mFI = 0), if experienced a major in-hospital complication: 54% vs 22%, *P* < 0.001 Frail patients vs robust, if not experienced a major in-hospital complication: 23% vs 7%, *P* < 0.001 Pre-frail patients (mFI 0.09–0.18) vs robust: OR 1.37 (95% CI 1.07–1.74), *P* = 0.01 Frail patients vs robust: OR 2.33 (95% CI 1.34–4.03), *P* = 0.003
Meng *et al*., 2018 [25]	Retrospective (ACS-NSQIP Database)	1516	RC	11-item mFI^a^	Discharge to a higher level of care vs at home	mFI: AUC 0.586 (0.536–0.636), did *not* reach statistical significance
Sathianathen *et al*., 2018 [27]	Retrospective (ACS-NSQIP Database)	5516	RC	5-item sFI^c^ and 11-item eFI^b^	Discharge to a facility vs home	Frail (sFI ≥3) and pre-frail patients (sFI = 2) vs non-frail (sFI = 0): 27.8% and 18.7% vs 8.4%, *P* < 0.001 Pre-frail patients vs non-frail: OR 1.54 (95% CI 1.18–2.02) Frail patients vs non-frail: OR 2.31 (95% CI 1.40–3.82) sFI: AUC 0.60 (95% CI 0.58–0.62) eFI: AUC 0.58, *P* = 0.5
Unplanned readmission						
Michel *et al*., 2019 [23]	Retrospective (NRD)	9459	ORC, RARC	Johns Hopkins ACG frailty-defining diagnoses indicator	30-day readmissions; Readmission costs	Frail patients vs non-frail, 30-day readmissions: 31.8% vs 29.3%, +Δ2.5% (95% CI – 4.2% to 9.2%), did *not* reach statistical significance. Frail patients vs non-frail, median (IQR) readmission costs: $35 732 ($26 638–$56 440) vs $29 319 ($22 314–$39 513)
Woldu *et al*., 2018 [29]	Retrospective (monocentric study)	346	ORC, RARC	11-item mFI^b^	90-day readmission	mFI: AUC 0.547 (95% CI 0.479–0.616), *P* = 0.2
Chappidi *et al*., 2016 [26]	Retrospective (ACS-NSQIP Database)	2679	RC	11-item mFI^b^	30-day readmission	Pre-frail and frail patients (mFI ≥2) vs other patients (mFI <2): 20.3% vs 21.1%, *P* = 0.7
Lascano *et al*., 2015 [21]	Retrospective (ACS-NSQIP Database)	41,681 (5709, 7791 1443, 23,350,3388)	PN, RN, RNU, RP, ORC	15-point mFI	Readmission rates	Frail patients (15-point mFI ≥0.20) vs non frail (15-point mFI 0–0.05): 15.9% vs 19.7%, *P* = 0.3
Burg *et al*., 2018 [31]	Prospective (monocentric study)	123, aged ≥65 years	ORC, RARC	FFC	30/90-day readmission rates	No preoperative assessment variables were significant for 30-day readmissions. Decreased gait speed was significantly associated with 90-day readmission (*P* = 0.02).
Early mortality rates						
Palumbo *et al*., 2019 [22]	Retrospective (NIS database)	23,967	ORC, LRC, RARC	Johns Hopkins ACG frailty-defining diagnoses indicator	In-hospital mortality	Frail patients vs non-frail: 2.4% vs 1.5%, *P* < 0.001 Frail patients vs non-frail: OR 1.45 (95% CI 1.17–1.80), *P* = 0.001
Michel *et al*., 2019 [23]	Retrospective (NDR)	9459	ORC, RARC	Johns Hopkins ACG frailty-defining diagnoses indicator	In-hospital mortality	Frail patients vs non-frail: 4.2% vs 1.5%, +Δ2.6%; 95% CI [0.1%-5.2%], p = 0.04 Frail patients vs non-frail: OR = 2.30, 95% CI (1.08–4.92), p = 0.03
Meng *et al*., 2018 [25]	Retrospective (ACS-NSQIP Database)	1516	RC	11-item mFI^a^	30-day mortality	mFI: AUC 0.537 (95% CI 0.453–0.621), did *not* reach statistical significance
Chappidi *et al*., 2016 [26]	Retrospective (ACS-NSQIP Database)	2679	RC	11-item mFI^b^	30-day mortality	Frail and pre-frail patients (mFI ≥2) vs other patients (mFI <2): 3.5% vs 1.8%, *P* = 0.01 Pre-frail patients (mFI = 2) vs non-frail (mFI = 0): OR 1.24 (95% CI 0.62–2.45), *P* = 0.6 Frail patients (mFI ≥3) vs non-frail: OR 2.07 (95% CI 0.78–5.49), *P* = 0.1
Taylor *et al*., 2019 [28]	Retrospective (ACS-NSQIP Database)	92,999 (3823, 8154, 14,668, 2817, 13,953, 5678, 9466)	MIRP, RRP, MIRN, ORN, MIPN, OPN, RC	5-item sFI^d^	30-day mortality	In RC patients: sFI: sFI = 0, 0.8%; sFI = 1, 1.5%; sFI = 2, 2.2%; sFI ≥3, 3.9%; *P* < 0.001
Lascano *et al*., 2015 [21]	Retrospective (ACS-NSQIP Database)	41,681 (5709, 7791 1443, 23,350, 3388)	PN, RN, RNU, RP, ORC	15-point mFI and 11-item mFI^a^	30-day mortality	In RC patients: Frail patients (15-point mFI ≥0.20) vs non frail (15-point mFI 0–0.05): 6.8% vs 2.1%, *P* = 0.005 15-point mFI: AUC 0.574, *P* < 0.001 The 15-point mFI was superior to the 11-item mFI in all the comparisons.

FTR: failure to rescue; LRC: laparoscopic RC; MIPN: minimally invasive PN; MIRN: minimally invasive RN; MIRP: minimally invasive RP.

^a^The mFI was calculated as the number of present factors divided by the total number of index factors. Patients were scored as: ‘robust’ (mFI = 0), ‘pre-frail’ (mFI 0.09–0.18), or ‘frail’ (mFI ≥0.27).

^b^The mFI was calculated by scoring the number of risk factors per patient: 0, 1, 2, and ≥3.

^c^The sFI was calculated by scoring the number of risk factors per patient: 0, 1, 2, and ≥3 (full score of 5).

^d^The sFI was calculated by scoring the number of risk factors per patient: 0, 1, 2, and ≥3 (full score of 6).

^e^Major complications as described by the ACS-NSQIP included coma for >24 h, stroke with residual deficits, unplanned intubation, ventilator requirement for >48 h, deep incisional surgical site infection, organ space surgical site infection, wound disruption, sepsis, septic shock, acute renal failure, progressive renal insufficiency, myocardial infarction, cardiac arrest requiring cardiopulmonary resuscitation, deep venous thrombosis, and pulmonary embolism.

#### Postoperative complication rates

The indicator that identified the greatest impact of frailty on early postoperative complication rates was the FFC, recording an almost five-fold increased risk of major complications within 30 days and three-fold within 90 days after RC for intermediately frail or frail patients compared to non-frail patients [31]. A similar impact was highlighted by the Johns Hopkins ACG indicator, which appeared to be valid especially in predicting the relationship between frailty and ICU-level complications (OR 4.74) [23], while the risk of overall complications for frail patients identified with this index was 1.5-times higher [22]. Using this index, 68% of frail patients were found to have a postoperative complication vs 56% of non-frail patients [22]; regarding ICU-level complications, the percentages were 53% vs 19% (all *P* < 0.001) [23]. The sFI demonstrated its ability to identify a risk more than three-times greater of 30-day major complications in patients diagnosed as frail and its non-inferiority to the extended 11-item index [27]. The impact of being frail on the risk of postoperative RC-related complications assessed by the mFI was lower than with the sFI (OR 2.58 vs 3.22), while for pre-frail patients the two results were closer (OR 1.84 vs 1.73) [26,27]. Using the sFI, 43% (range 26–60%) of frail and 35% (range 15–55%) of pre-frail patients had a major complication vs 27% (range 8–45%) of non-frail patients (all *P* < 0.001) [27,28], while based on the mFI the percentage of frail and pre-frail patients who had a major complication was 23% (range 15–32%) vs 8.6% (range 8.3–8.8%) of non-frail patients (all *P* < 0.001) [24,26]. The sFI, moreover, confirmed an approximately three-fold increase in the likelihood of postoperative complications even in a subpopulation of octogenarians [30]. In two cases the mFI did not achieve statistical significance [25,29]. The 15-point mFI was found to have poor predictive value in identifying the risk of severe complications (CCS = IV) for patients undergoing RC (AUC 0.585, *P* < 0.001), although its ability seemed to exceed that of the 11-item mFI [21].

#### LOS and costs

The greatest difference in LOS between frail and non-frail patients was highlighted by the Johns Hopkins ACG indicator, showing an almost doubled LOS [23]. The mFI also revealed a prolongation of LOS in patients diagnosed as frail, but the impact was less evident [24,26,29] and in one study this index did not achieve statistical significance [25]. Frailty detected with the sFI was not significantly associated with LOS prolongation in an octogenarian cohort [30].

In terms of hospitalisation costs, the impact of frailty in increasing charges measured with the Johns Hopkins ACG indicator was more evident than that identified by the mFI [23,29].

#### Discharge disposition

The Johns Hopkins ACG indicator showed that frailty increased the risk of non-home discharge by almost 3.5 times, identifying the highest impact on this outcome [23]. Using this indicator, 34% of frail patients were discharged non-home vs 12% of non-frail patients (*P* < 0.001) [23]. The mFI and sFI showed to be significantly associated with an increased risk of discharge other than to home with a comparable impact in both pre-frail (OR 1.37 vs 1.54) and frail patients (OR 2.33 vs 2.31) [24,27]. Based on the sFI, 28% of frail and 19% of pre-frail patients were discharged to a facility rather than home vs 8% of non-frail (*P* < 0.001) [27]. In one study the relationship between frailty and discharge disposition identified by the mFI was not statistically significant [25].

#### Unplanned readmission

None of the in-studio indicators achieved significance in predicting the influence of frailty on the risk of readmission after RC. Only a decreased gait speed (a criterion of the FFC) was significantly associated with the 90-day readmission rate [31].

#### Early mortality rates

The Johns Hopkins ACG indicator was shown to be a predictor of in-hospital mortality by identifying that the risk of dying before being discharged increased more than twice for frail patients compared to the others [23]. Using this indicator, 3% (range 2–4%) of frail patients died during their in-hospital stay vs 1.5% of non-frail patients (*P* < 0.05) [22,23]. The risk of 30-day mortality increased significantly with rising levels of frailty identified by the sFI: according to this index, 4% of frail and 2% of pre-frail patients died within 30 days after RC vs 1% of non-frail (*P* < 0.001) [28]. The mFI did not achieve or did not confirm in statistical analyses the significance in the detection of this association [25,26]. The 15-point was superior to the mFI, although its 30-day mortality predictive ability remained low for RC patients (AUC 0.574, *P* < 0.001) [21]. According to this index, the percentage of frail patients who died within 30 days after RC was 7% vs 2% of non-frail patients (*P* < 0.001).

## Discussion

Patients frailty at baseline has been associated with a higher rate of adverse postoperative events in several types of surgical procedures [10]. Similarly, preliminary results were recently presented in the management of urological malignancies [32,33]. Despite the progress in the quality of care, RC still carries a heavy burden of unfavourable outcomes [5,6]. Therefore, the preoperative assessment of the risk factors for postoperative complications and early mortality could help clinicians in providing tailored counselling, selecting the best treatment, and refining a personalised care pathway. We performed a systematic review to assess the prevalence of frailty in patients with BCa undergoing RC and summarise the current evidence regarding the prognostic value of frailty measurements on early postoperative outcomes.

We found that 8% (range 2–24%) of patients undergoing RC were diagnosed as frail and 31% (range 14–65%) as pre-frail, a condition used to describe patients diagnosed with some components of a frailty measure but not enough to meet the defined frailty cut-off [11]. Considering only octogenarians, frail and pre-frail patients increased to >60% [30]. These results underline the importance of focussing on the preoperative identification of frailty in patients with BCa undergoing RC, a population with a high proportion of subjects particularly vulnerable to the stressors associated with this major surgery. Our present findings are consistent with a recent meta-analysis of frailty in general surgery, in which frail patients proportion ranged between 10% and 37% and that of pre-frail between 31% and 46% [34]. This wide variability of prevalence was probably due to differences in frailty assessment methods and limitations inherent in retrospective observational studies.

Postoperative complications were the most commonly studied and reported outcomes in the articles reviewed (11 out of 11 studies). Moreover, the strongest evidence of association was found between frailty and 30-day major complications (CCS ≥III) and between frailty and the risk of in-hospital complications requiring ICU management (CCS = IV), that was up to five-times increased in frail patients and almost doubled in pre-frail patients. These associations were consistent across different frailty measurements. The impact of frailty on postoperative complications rate appeared stronger than advanced age (≥80 years), obesity, smoking habit, history of a bleeding disorder, and being underweight. Furthermore, frailty was associated with increased early mortality rates, proving to be a significant risk factor for both in-hospital mortality (frail patients had a more than doubled risk than non-frail, according to the John Hopkins index), and for mortality within 30 days after surgery (frail patients had a likelihood almost four-times higher than non-frail, based on the sFI). Our present findings were congruent with other reviews about the role of frailty in surgical patients [14,35], and were consistent with analyses of urological oncological surgery [32], as well as with previous reviews on RC [12,36]. All these studies demonstrated frailty to be significantly associated with early mortality and postoperative complications. As highlighted by Chappidi *et al*. [26], the cut-off separating frail and pre-frail patients from the others represented a threshold beyond which high-grade complications and mortality rates significantly increased. Therefore, this cut-off could provide clinical utility in identifying those patients for whom it is reasonable to consider other potential treatment options as an alternative to surgery.

The collected results showed that frailty was significantly related to a longer median LOS and increased hospitalisation costs. It seemed, on the other hand, that frailty was not independently associated with the readmission rate. However, Taylor *et al*. [28] showed that RC was the largest contributor, among several urological procedures, to the healthcare resource utilisation (HRU), a comprehensive outcome that unified pLOS (>75th percentile), discharge to continued care, and 30-day unplanned readmission. It can, therefore, be reasonably inferred that a frail status may affect the readmission risk after RC, although this topic should be more thoroughly investigated.

Moreover, we found that frail patients had a more than tripled risk of being discharged to a rehabilitation or nursing facility rather than home compared to non-frail patients; the results for pre-frail patients were similar. Pearl *et al*. [24] observed that this likelihood was significantly higher for frail and pre-frail patients than for non-frail, whether they experienced a major in-hospital complication (54% vs 22%) or they did not (23% vs 7%). Thus, the authors suggested that ongoing efforts to minimise these complications among frail patients may not impact their ultimate discharge disposition. This finding, indeed, highlighted how important it is not only to prevent complications, but also to ensure the best possible postoperative course for these patients by avoiding common risk factors (e.g. delirium and falls) that are related to non-home discharge. The evidence showed that a higher proportion of patients discharged to a location other-than-home experienced early mortality after RC compared to those discharged home [24,37,38]. Therefore, still bearing in mind the possible risk of selection bias related to the frail population, the increased rate of non-home discharge among frail patients undergoing RC could submit them to this additional risk of short-term mortality.

We focussed on the type of index used to assess frailty and we found that the mFI and sFI were the most widely employed in the literature (in four and three of the 11 studies, respectively). Comparing the different methods of frailty measurement, we found that the Johns Hopkins ACG indicator proved to be a reliable predictor of in-hospital mortality and demonstrated the best ability to predict the likelihood of non-home discharge after RC and the risk of severe postoperative complications, distinguishing itself as one of the best prognostic measurements of frailty’s impact on the RC population [22,23]. Among the other frailty indexes, the five-item sFI appeared the most suitable predictor of postoperative RC-related complications and discharge other than home, as it demonstrated a good prognostic ability and is also easier to apply than other indicators. Moreover, the comparable predictive ability of the sFI and 11-item mFI (or eFI) suggested that there is no compromise in accuracy when using the simpler method [27]. Both these indexes showed an ability to predict the risk of postoperative major complications, pLOS and non-home discharge that overlapped or even exceeded that of conventional risk scores (ASA score and CCI). These results should further promote the use of frailty indexes in clinical practice for preoperative patient assessment together with other already widely used indexes. The 15-point mFI combines items related to comorbidity and functional status with oncological variables, thus increasing the accuracy in describing the patient’s conditions. It was found to be superior to the 11-item mFI in both predicting the risk of postoperative severe complications (CCS = IV) and 30-day mortality [21]; however, its predictive ability, which was valuable for other urological interventions (RP, PN, RN, and RNU) appeared poor for RC and should be deepened in targeted studies. From our present findings, the FFC proved to be a promising but not yet widely used assessment method to identify the patients most at risk of postoperative major complications, showing the highest impact of frailty in increasing the risk of this outcome within 30 days after RC [31].

According to our present results, fewer frail compared to non-frail patients undergo RC with a minimally invasive approach (14% vs 17%) [22,23,29]. However, in a recent retrospective study comparing the impact of frailty on ORC vs RARC, time trends revealed an increased rate of RARC among frail patients (+27% between 2000 and 2015), while ORC rates remained stable over the time [39]. Considering that frail patients are frequently malnourished and express higher levels of inflammatory markers, hyperactivation of the coagulation system and altered metabolism [40,41], they would be those most likely to benefit from a robot-assisted approach, due to its intrinsic minimal invasiveness, reduced surgical stress, tissue trauma, and systemic inflammation [42]. On the other hand, recent evidence showed that the use of RARC in the frail population did not result in better short-term outcomes except for a 1-day advantage in LOS: consequently, the RARC vs ORC benefit appeared relatively marginal in frail compared to non-frail patients, whereas, in as previously mentioned, this approach resulted in lower complication rates and shorter LOS [39]. To explain this difference, it should probably be taken into consideration that the full benefits of RARC have been found mainly through the use of an intracorporeal approach [43], and this type of reconstruction tends to be applied less in frail and elderly patients, due to its higher rates of overall complications and readmissions compared to extracorporeal UD [44]. Further and prospective studies are needed to test these topics, considering also that, to date, the evidence has failed to show a quality of life benefit of RARC compared to ORC [45].

Determining before surgery the frailty index of each patient and the potential early postoperative effect of RC based on his/her frailty status could have important clinical implications. This would allow clinicians to provide personalised counselling, implement the correct surgical techniques, plan an adequate post-surgical course and post-discharge pathway or even modify the indication for surgery, offering potential alternatives (chemotherapy, radiotherapy, or bladder-preserving trimodal therapy). Furthermore, combining frailty assessment with a validated preoperative risk grouping [46], and with the indications provided by an international collaborative consensus [47] would increase the accuracy in selecting patients most likely to benefit from neoadjuvant chemotherapy. Patients identified as frail could, also, profit from strategies aimed to optimise their condition and consequently their postoperative and survival outcomes. In this regard, the application of *prehabilitation* [48], as well as immunonutrition protocols [49] is becoming increasingly interesting. Furthermore, the principles of the ERAS programmes have been shown to be particularly well suited for elderly and compromised patients [50].

Our present systematic review has some limitations. First, the studies collected were mostly retrospective and based on single-centre cohorts or national databases. Therefore, the results may have been exposed to selection bias or bias due to missing data. Second, the wide variety of frailty definitions and cut-offs, as well as the use of different statistical methods, were limitations for cross-study comparisons; comparative prospective trials would be useful to determine the best index in both predictive ability and ease-of-use in preoperative settings. Third, our research was limited to English-language records and this may have affected the choice of eligible items.

## Conclusion

About 40% of the patients with BCa undergoing RC are frail or pre-frail. Frailty was predictive of an increased likelihood of early postoperative major complications, non-home discharge, longer LOS, higher costs, and early mortality. Among the measures assessing frailty in the RC population the most used index was the mFI, while the John Hopkins indicator and sFI were found to be the most reliable indexes to identify patients at greater risk of postoperative RC-related adverse events. Preoperative frailty evaluation should be routinely included in clinical practice to improve surgical decision-making among clinicians, patients, and their families and to optimise early postoperative outcomes. Further prospective comparative studies are required to gain a better standardisation of frailty cut-offs and measurements.

## Supplementary Material

Supplemental Material
