# Supplementary material for: Frailty impact on postoperative complications and early mortality rates in patients undergoing radical cystectomy for bladder cancer: a systematic review
Source: Arab J Urol. 2020 Nov 2;19(1):9–23. doi: 10.1080/2090598X.2020.1841538 (PMC7954492; doi:10.1080/2090598X.2020.1841538)
Supplement: Supplemental Material [file TAJU_A_1841538_SM4224.docx]

Supplementary Table S1. Overview of the studies investigating the relationship between preoperative frailty and early postoperative RC-related outcomes.

| Author,  year | Study design | Study size, *n* | Type of surgery | Preoperative frailty indicator(s) | Postoperative outcomes | Findings |
| --- | --- | --- | --- | --- | --- | --- |
| Palumbo *et al.*, 2019 [22] | Retrospective (NIS database) | 23967 | ORC, LRC, RARC | Johns Hopkins ACG frailty-defining diagnoses indicator | Overall complications;  FTR;  In-hospital mortality;  pLOS;  THCs | Prevalence of frail patients: 24.3%  Frail patients were more likely to experience in-hospital mortality (2.4% vs 1.5%; *P* < 0.001) and overall complications (67.9% vs 55.8%, *P* < 0.001) compared to non-frail patients.  Frailty was an independent predictor of overall complications (OR 1.54, 95% CI 1.44–1.65; *P* < 0.001), FTR (OR 1.64, *P* < 0.001), in-hospital mortality (OR 1.45, 95% CI 1.17–1.8; *P* = 0.001), pLOS (RR 1.32, 95% CI 1.28–1.35; *P* < 0.001), THCs (+$8003.3, 95% CI $6849.1–$9158.2; *P* < 0.001). The magnitude of its association was stronger or at least equal than that of age ≥75 years and CCI ≥2. |
| Michel *et al.*, 2019 [23] | Retrospective (NRD) | 9459 | ORC, RARC | Johns Hopkins ACG frailty-defining diagnoses indicator | In-hospital mortality;  ICU-level complications (CCS = IV);  pLOS;  30-day readmission;  Non-home discharge;  Hospital-related costs | Prevalence of frail patients: 7.1%  Frail patients were more likely to experience in-hospital mortality (4.2% vs 1.5%; +Δ2.6%, 95% CI 0.1–5.2%; *P* = 0.04), ICU-level complications (52.9% vs 18.6%; +Δ34.3%, 95% CI 30.0–40.6%; *P* < 0.001) and non-home discharge (33.9% vs 11.6%; +Δ22.2%, 95% CI 16.2–14.5%; *P* < 0.001) compared to non-frail.  Frailty was an independent predictor of ICU-level complications (OR 4.74, 95% CI 3.60–6.25; *P* < 0.001), pLOS (OR 0.58, 95% CI 0.50–0.66; *P* < 0.001), non-home discharge (OR 3.43, 95% CI 2.50–4.69; *P* < 0.001), hospital-related costs (OR 0.42, 95% CI 0.34–0.49; *P* < 0001) and in-hospital mortality (OR 2.30, 95% CI 1.08–4.92; *P* = 0.03). The magnitude of its association was comparable or even stronger than that of age ≥75 years and CCI ≥3.  Frailty was not independently associated with 30-day readmission. |
| Pearl *et al.*, 2017 [24] | Retrospective (ACS-NSQIP Database) | 4330 | RC | 11-item mFI^a^ | Discharge disposition;  Any in-hospital complication;  Major in-hospital complications^e^;  LOS;  30-day readmission;  30-day mortality | Prevalence of frail patients: 2.2%; and of pre-frail patients: 65.1%.  Both frail and pre-frail patients were more likely to be discharged to non-home care vs robust (OR 2.33, 95% CI 1.34–4.03, *P* = 0.003 and OR 1.37, 95% CI 1.07–1.74, *P* = 0.01, respectively).  mFI ≥0.27 was significantly related to a higher proportion of any (28.7% vs 19.6%, *P* < 0.001) and major (19.2% vs 8.8%, *P* < 0.001) in-hospital complication and with a pLOS, at 9 (4–57) vs 7 (0–75) days (*P* < 0.001).  If frail patients had a major in-hospital complication, they had 54% probability of non-home discharge vs 22% for robust patients (*P* < 0.001), if they did not have a major complication the proportion was: 23% for frail vs 7% for robust (*P* < 0.001).  A higher proportion of patients discharged to a location other than home had 30-day mortality compared with those that were discharged home (2.17% vs 0.56%, *P* < 0.001).  There was no difference in the readmission rate of those patients discharged to non-home-based vs home-based care (17.73% vs 17.54%, *P* = 0.87) |
| Meng *et al*., 2018 [25] | Retrospective (ACS-NSQIP database) | 1516 | RC | 11-item mFI^a^ | 30-day adverse events;  pLOS (>75th percentile);  30-day mortality;  Discharge to a higher level of care | Prevalence of frail patients: 7.4%  mFI (AUC 0.500, 95% CI 0.471–0.529) vs mCCI (AUC 0.511, 95% CI 0.485–0.537) and ASA score (AUC 0.510, 95% CI 0.485–0.534) had the worst performance at predicting any adverse events and did *not* reach statistical significance.  mFI was not predictive of serious adverse events, pLOS, 30-day mortality and discharge to a higher level of care. |
| Woldu *et al.*, 2018 [29] | Retrospective (monocentric study) | 346 | ORC, RARC | 11-item mFI^b^ | 30-day major complications (CCS ≥III);  90-day readmission;  LOS;  Hospital-related costs | Prevalence of frail patients: 16.7%; and pre-frail patients: 24%  Analysis showed a weak association of all indices with major complications after RC: AUC 0.535 for the ASA class; 0.565 for the CCI score; and 0.551 (95% CI 0.471–0.631, *P* = 0.2) for the mFI.  There was a weak association of all indices with readmission rate too.  mFI = 2 and mFI ≥3 were predictors of pLOS [mean (SD) 8.2 (5.7) and 11.3 (11.1) vs 7.6 (4.7) days, *P* = 0.003] compared to mFI = 0, while neither ASA score nor CCI reached statistical significance.  Associated costs were significantly higher in frail patients than in the other groups at $30 354 vs ~$22 500 (*P* = 0.003) |
| Chappidi *et al.*, 2016 [26] | Retrospective (ACS-NSQIP database) | 2679 | RC | 11-item mFI^b^ | 30-day overall complications;  30-day severe complications (CCS IV–V);  LOS;  30-day readmission;  30-day ROR;  30-day mortality | Prevalence of frail patients: 4%; and pre-frail patients: 21%.  Patients with mFI ≥2 had a higher proportion of severe complications (14.6% vs 8.3%, *P* < 0.001) and overall mortality (3.5% vs 1.8%, *P* = 0.01) within 30 days after RC and had a pLOS [median: 11.0 (10.2–11.8) vs 10.0 (9.7–10.4), *P* < 0.01]. There was no difference in overall complication rates, 30-day readmission, and 30-day ROR rates between patients with mFI <2 and mFI ≥2.  On multivariable analyses, mFI = 2 (OR 1.84, 95% CI 1.28–2.64; *P* = 0.001) and mFI ≥3 (OR 2.58, 95% CI 1.47–4.55; *P* = 0.001) were independent predictors of 30-day severe complications, while mFI was not significantly related to 30-day overall mortality. |
| Sathianathen *et al.*, 2018 [27] | Retrospective (ACS-NSQIP database) | 5516 | RC | 5-item sFI^c^ and 11-item eFI^b^ | 30-day major complications (CCS ≥III);  Discharge destination | Prevalence of frail patients: 2.2%; and of pre-frail patients: 19.9%.  The complication rate was 7.7%, 10.3%, 15.1%, and 26.0% for sFI scores of 0, 1, 2, and ≥3, respectively (*P* < 0.001). The percentage of patients discharged to a facility was 8.4%, 13.9%, 18.7%, and 27.8% for sFI scores of 0, 1, 2, and ≥3, respectively (*P* < 0.001).  sFI = 2 (OR 1.73, 95% CI 1.32–2.26) and an sFI ≥3 (OR 3.22, 95% CI 2.01–5.17) patients had a greater likelihood of having a major complication.  sFI = 2 (OR 1.54, 95% CI 1.18–2.02) and sFI ≥3 (OR 2.31 95% CI 1.40–3.82) were associated with an increased likelihood of being discharged to a facility.  In ROC curve analyses, sFI outperformed the ASA score in predicting major complications (AUC 0.561 vs 0.544) and its predictive ability was comparable to the more complex eFI and to NSQIP risk calculator.  sFI was a comparable predictor of non-home discharge as both the ASA score (*P* = 0.4) and eFI (*P* = 0.5). |
| De Nunzio *et al.*, 2019 [30] | Retrospective (multicentre study) | 117, aged ≥80 years | RC | 5-item sFI^c^ | 90-day major complications (CCS ≥III);  Postoperative bowel canalization;  LOS | Prevalence of frail patients: 38.5%; and pre-frail patients: 26.5%.  sFI ≥3 was an independent risk factor of major complications (OR 3.10, 95% CI 0.70–13.70; *P* = 0.01) compared to sFI <3.  No significant differences were detected in terms of LOS (*P* = 0.28) and postoperative bowel canalisation (*P* = 0.41) when related to sFI. |
| Taylor *et al.*, 2019 [28] | Retrospective (ACS-NSQIP database) | 92999 (3823,8154, 14668, 2817, 13953, 5678, 9466) | MIRP, RRP, MIRN,  ORN, MIPN, OPN, RC | 5-item sFI^d^ | HRU:  1. discharge to continued care,  2. 30-day readmission,  3. pLOS (>75th percentile);  30-day any complications;  30-day major complication^e^  30-day mortality | Prevalence of frail patients in the whole cohort: 4.6%; and of pre-frail patients: 12%.  Prevalence of frail patients in RC cohort: 6.7%; and of pre-frail patients: 14,2%  Increased sFI was significantly associated with increased HRU, relative to sFI = 0, in all types of surgery (*P* < 0.001): sFI = 1, OR 1.20 (95% CI 1.16–1.25); sFI = 2, OR 1.53 (95% CI 1.45–1.61); sFI ≥3, OR 1.95 (95% CI 1.81–2.09).  The most morbid procedure was RC with a statistically significant increase in the rate of any complication (sFI = 0, 50.8%; sFI = 1, 54%; sFI = 2, 61.2%; and sFI ≥3, 66.4%; *P* < 0.001) and major complications (45.3%; 49.0%; 55.3%; 60.3%, respectively; *P* < 0.001) together with sFI.  For RC patients, an increasing sFI was associated with increased proportions of 30-day mortality (sFI = 0, 0.8%; sFI = 1, 1.5%; sFI = 2, 2.2%; sFI ≥3, 3.9%; *P* < 0.001).  The largest contributor to HRU was RC, for which the risk of any HRU was 38% for sFI = 0 vs 56% for sFI ≥3. |
| Lascano *et al.*, 2015 [21] | Retrospective (ACS-NSQIP database) | 41681 (5709, 7791 1443, 23350, 3388) | PN, RN, RNU, RP, ORC | 15-point mFI and 11-item mFI^a^ | 30-day CCS =IV complications;  30-day mortality;  ROR rates;  Readmission rates | Prevalence of frail patients in RC cohort: 5.2%  Frail patients (15-point mFI ≥0.20) had a higher risk of 30-day CCS = IV complications (OR 3.70, 95% CI 2.87–4.79; *P* < 0.001) and increased risk of 30-day mortality (OR 5.95, 95% CI 3.72–9.51; p=0.0005) in comparison with non-frail patients (15-point mFI 0–0.05) in all types of surgery.  For RC, being frail was associated with an increased risk of 30-day CCS = IV complications (17.0% vs 6.6%, *P* < 0.001) and 30-day mortality (6.8% vs 2.1%, *P* = 0.005) compared to not being frail.  15-point mFI was not a predictor of ROR or readmission for RC patients.  For RC, 15-point mFI compared with ASA score was a poor predictor of 30-day CCS = IV complications (AUC 0.585 vs 0.612, *P* < 0.001) and 30-day mortality (AUC 0.574 vs 0.612, *P* < 0.001).  The 15-point mFI was superior to the 11-item mFI in all the comparisons. |
| Burg *et al.*, 2018 [31] | Prospective (monocentric study) | 123, aged ≥65 years | ORC, RARC | FFC | 30/90-day any complications;  30/90-day major complications (CCS ≥III);  30/90-day readmission rates | Prevalence of frail patients: 5.5%; and of intermediately frail: 39.5%  Shrinking was an independent predictor of any 30-day complication (OR 3.79, 95% CI 1.64–9.26; *P* = 0.002), while physical activity was protective for both 30-day major complications (OR 0.36, 95% CI 0.12−0.78; *P* = 0.04) and any 90-day complication rate (OR 0.84, 95% CI 0.69–1.00; *P* = 0.03).  Being intermediately frail or frail was associated with both 30-day (OR 4.87, 95% CI 1.39–22.77; *P* = 0.02) and 90-day major complications rates (OR 3.01, 95% CI 1.05–9.37; *P* = 0.04).  No preoperative assessment variables were significant for 30-day readmissions.  Decreased gait speed (*P* = 0.02) was significantly associated with 90-day readmission. |

ACG: Adjusted Clinical Groups; ACS: American College Surgeons; AUC: area under the curve; CCI: Charlson Comorbidity Index; CCS: Clavien-Dindo Classification System; eFI: extended Frailty Index; FFC: Fried Frailty Criteria; FTR: failure to rescue; HRU: health care resource utilisation; ICU: intensive care unit; (p)LOS: (prolonged) length of hospital stay; LRC: laparoscopic radical cystectomy; NSQIP: National Surgical Quality Improvement Program; OPN: open partial nephrectomy; ORC: open radical cystectomy; mFI: modified Frailty Index; MIPN: minimally invasive partial nephrectomy; MIRN: minimally invasive radical nephrectomy; MIRP: minimally invasive radical prostatectomy; NIS: National Inpatient Sample; NRD: Nationwide Readmissions Database; OR: odds ratio; ORN: open radical nephrectomy; PN: partial nephrectomy; RARC: robot-assisted radical cystectomy; RC: radical cystectomy; RN: radical nephrectomy; RNU: radical nephroureterectomy; ROC: receiver operating characteristic; ROR: return to the operating room; RP: radical prostatectomy; RR: relative risk; RRP: radical retropubic prostatectomy; sFI: simplified Frailty Index; SSI: surgical site infection; THCs: total hospital charges.

^a^ The mFI was calculated as the number of present factors divided by the total number of index factors. Patients were scored as: ‘robust’ (mFI = 0), ‘pre-frail’ (mFI 0.09–0.18), or ‘frail’ (mFI ≥0.27).

^b^ The mFI was calculated by scoring the number of risk factors per patient: 0, 1, 2, and ≥3.

^c^ The sFI was calculated by scoring the number of risk factors per patient: 0, 1, 2, and ≥3 (full score of 5).

^d^ The sFI was calculated by scoring the number of risk factors per patient: 0, 1, 2, and ≥3 (full score of 6).

^e^ Major complications as described by the ACS-NSQIP included coma for >24 h, stroke with residual deficits, unplanned intubation, ventilator requirement for >48 h, deep incisional surgical site infection, organ space surgical site infection, wound disruption, sepsis, septic shock, acute renal failure, progressive renal insufficiency, myocardial infarction, cardiac arrest requiring cardiopulmonary resuscitation, deep venous thrombosis, and pulmonary embolism.
